# Application of Contrast-Enhanced Ultrasound in Cystic Pancreatic Lesions Using a Simplified Classification Diagnostic Criterion

**DOI:** 10.1155/2015/974621

**Published:** 2015-05-18

**Authors:** Zhihui Fan, Kun Yan, Yanjie Wang, Jianxing Qiu, Wei Wu, Lei Yang, Minhua Chen

**Affiliations:** ^1^Key Laboratory of Carcinogenesis and Translational Research (Ministry of Education), Department of Ultrasound, Peking University Cancer Hospital & Institute, No. 52, Fucheng Road, Haidian District, Beijing 100142, China; ^2^Department of Radiology, Peking University First Hospital, No. 8, Xishiku Street, Xicheng District, Beijing 100034, China; ^3^Key Laboratory of Carcinogenesis and Translational Research (Ministry of Education), Department of Beijing Office for Cancer Prevention and Control, Peking University Cancer Hospital & Institute, No. 52, Fucheng Road, Haidian District, Beijing 100142, China

## Abstract

*Objective.* Classification diagnosis was performed for cystic pancreatic lesions using ultrasound (US) and contrast-enhanced ultrasound (CEUS) to explore the diagnostic value of CEUS by comparison with enhanced CT.* Methods*. Sixty-four cases with cystic pancreatic lesions were included in this study. The cystic lesions of pancreas were classified into four types by US, CEUS, and CT: type I unilocular cysts; type II microcystic lesions; type III macrocystic lesions; and type IV cystic lesions with solid components or irregular thickening of the cystic wall or septa.* Results*. Eighteen type I, 7 type II, 10 type III, and 29 type IV cases were diagnosed by CT. The classification results by US were as follows: 6 type I; 5 type II; 4 type III; and 49 type IV cases. Compared with the results by enhanced CT, the kappa value was 0.36. Using CEUS, 15, 6, 12, and 31 cases were diagnosed as types I–IV, respectively. The kappa value was 0.77.* Conclusion*. CEUS has obvious superiority over US in the classification diagnostic accuracy in cystic pancreatic lesions and CEUS results showed substantial agreement with enhanced CT. CEUS could contribute to the differential diagnosis of cystic pancreatic diseases.

## 1. Introduction

With the development and extensive application of imaging techniques, the rate of detecting cystic pancreatic lesions is increasing. These cystic lesions may be epithelium-derived tumors or formed from the necrosis and cystic degeneration of solid tumors. The clinical treatment schemes and prognosis vary for different cystic lesions. Therefore, imaging diagnosis has significant clinical value. Currently, CT and MRI are still the major tools used in the differential diagnosis and follow-up of cystic pancreatic lesions.

In recent years, contrast-enhanced ultrasound (CEUS) has been increasingly used in the evaluation of pancreatic lesions. A study [1] has shown that the diagnostic accuracy of CEUS is comparable to that of MRI in the detection of septa and mural nodules in cystic pancreatic lesions. The correlation between CEUS findings and pathologic results was significantly stronger than between the sonographic and pathologic results. Previous studies involving CEUS in cystic pancreatic lesions have most often focused on the display of the components and nodules in cystic lesions as well as the enhancement features [2–4]. However, the different types of cystic pancreatic lesions cannot be easily differentiated due to the similar morphologic features. There are no simple and uniform diagnostic standards for CEUS. According to previous literature [5], an imaging classification system for cystic pancreatic lesions has been proposed and various types corresponding to different diseases had certain regularity. The classification on the basis of their imaging morphologic features is simple and easy to grasp and carry out. Further, such classification of cystic pancreatic lesions is helpful in characterizing lesions, narrowing the differential diagnosis [5]. In current study, the classification criterion was applied in US and CEUS for the first time. The application value of CEUS compared to US in the classification diagnosis of cystic pancreatic lesions is discussed.

## 2. Materials and Methods

### 2.1. Patients

Sixty-four patients with cystic pancreatic lesions who underwent CEUS and CECT examinations in our hospital between January 2007 and July 2013 were included. These patients were confirmed by surgery, biopsy, or comprehensive clinical diagnosis. Among the 64 patients, 23 underwent enhanced MRI simultaneously.

The patients included 43 females and 21 males, 22–80 years of age. The mean age was 52.4 years. Thirty-six patients had no symptoms and were identified by routine physical examination; 28 patients presented with various clinical symptoms. The major symptoms were abdominal distention, abdominal pain, fatigue, poor appetite, and emaciation. Only one patient had jaundice. Of 62 patients with CA19-9 results, 50 were normal and 12 showed elevations (42.08–1021 U/mL).

Forty-five cases were confirmed by pathologic examination of the surgical specimens, two were diagnosed by biopsy, and 17 by comprehensive clinical diagnosis. The standards for comprehensive diagnosis were as follows: the cases diagnosed by CT/MRI were followed up for >1 year and showed no obvious progression; the cases with unilocular cysts and a history of pancreatitis, without septa, calcifications, or mural nodules were diagnosed as pseudocysts; serous cystic adenomas (SCNs) had features of lobulated multilocular cysts without mural nodules or solid components, with microcysts <2 cm in diameter and a honeycomb shape, showing central calcifications or scarring by CT/MRI; intraductal papillary mucinous neoplasms (IPMNs) were diagnosed when unilocular or multilocular cystic lesions were observed by CT/MRI and the lesions communicated with the pancreatic duct; and all other cases with no typical manifestations and no changes in follow-up surveys were benign. CT-based classification diagnosis was considered the gold standard for evaluating the accuracy of US and CEUS.

### 2.2. US and CEUS

US and CEUS were performed by an ultrasound physician with over 10 years of experience in pancreas. The ultrasound instruments were GE Logiq 9 and GE Logiq E9 (GE Healthcare, Milwaukee, WI, USA); the probe frequency was 2.5–5.0 MHz; and the mechanical index was 0.12. The pancreas was first scanned by conventional ultrasonography to check for the position and size of the lesion and whether or not there were septa, calcifications, and dilated pancreatic ducts. Subsequently the CEUS was performed using SonoVue (Bracco Milan, Italy) as the contrast agent. Lyophilized SonoVue powder was dissolved in 5 mL saline. Two milliliters of the suspension was used for each examination and injected via the antecubital vein within 2–3 s, followed by a 5 mL saline flush. Real-time observation of the lesion should be no less than 120 s. Dynamic images were preserved for later analysis. Written informed consent was obtained from all the patients before the examinations. No adverse reactions occurred after CEUS. The study was approved by our institutional review board.

### 2.3. Image Analysis

**Figure 1:**
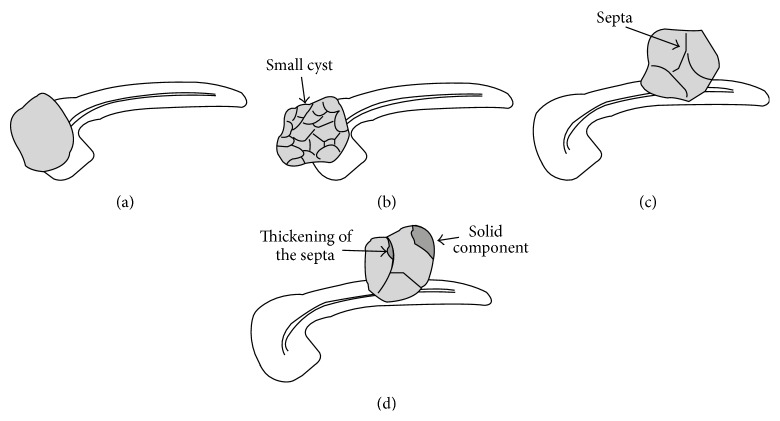
Schematic diagram of the four morphologic types of cystic pancreatic lesions. (a) Type I unilocular cyst. (b) Type II microcystic lesion. (c) Type III macrocystic lesion. (d) Type IV cystic lesions with solid components or irregular thickening of the cystic wall or septa.

Lesions were diagnosed separately by two ultrasound physicians with at least 5 years of CEUS experience without knowing in advance about the pathological or clinical diagnosis. Classification diagnosis was performed according to the morphologic features of the cystic pancreatic lesions [5] (Figure 1). Type I lesions are unilocular cysts (unilocular cysts without septa, solid components, or central and cystic wall calcifications, and the cystic wall is thin and uniform). Type II are microcystic lesions (the lesions are composed of several microcysts, which are a few millimeters to 2 cm in size, and central calcifications or scarring may be noted). Type III are macrocystic lesions (there were fewer compartments and the individual compartments had diameters >2 cm, and cystic wall calcifications may be visualized). Type IV are cystic lesions with solid components (unilocular or multilocular containing solid components, or the cystic wall and/or the septa are irregularly thickened, and the maximum thickness is ≥3 mm). When multiple lesions in one patient were observed, the diagnosis was made according to the largest lesions.

During CEUS, the first 30 s of CEUS is defined as the early stage of enhancement, while 31–120 s is defined as the late stage. It was observed whether or not the capsule and the septa of the lesions were enhanced as well as the degree of enhancement of the solid components. Designating the surrounding pancreatic parenchyma as a control, when the enhancement degree of the solid components was higher, equal to, and lower than the normal surrounding pancreatic parenchyma, the components were considered hyper-, iso-, or hypoenhancements. In cases in which there was divergence of opinions over the diagnostic results, a uniform diagnosis was made after a discussion between two physicians.

### 2.4. Enhanced CT

GE Lightspeed 64-slice spiral CT scan was applied with a slice thickness of 5 mm, interlayer spacing of 5 mm, a pitch of 1.5 or 2, and a voltage of 120 kV. Ninety milliliters (300 mgI/mL) of the nonionic contrast agent Iohexol was injected through the antecubital vein using a high-pressure injector with an injection speed of 3.5 mL/s. The arterial phase scan started 25–30 s after the injection, followed by a venous phase scan 30–35 s later. Two radiologists with >5 years of experience in the diagnosis of pancreatic lesions interpreted the CT images independently. The two radiologists did not know the results of CEUS or the pathologic and clinical diagnoses. The classification standards for the cystic lesions were the same as above (types I–IV). Any divergence was resolved by discussion.

### 2.5. Statistical Analysis

The statistical analysis was performed using SPSS 13.0. The measurement data were expressed as the mean ± SD. Dunnett* T3* test was applied in pairwise comparison between groups. A comparison of the US- and CEUS-based diagnoses with enhanced CT was performed using a chi-squared test. The concordance with the CT typing results was detected by kappa testing. The agreement was graded as follows: no agreement (0), slight (0–0.20), fair (0.21–0.40), moderate (0.41–0.60), substantial (0.61–0.80), and perfect agreement (0.81–1). The difference was considered statistically significant at a *P* < 0.05.

## 3. Results

### 3.1. Final Diagnosis

Of 64 patients, 61 had single lesions. Twenty lesions were located in the head of the pancreas or uncinate process, 18 in the pancreatic body, and 23 in the pancreatic tail. Three patients had multiple pancreatic lesions. The maximum diameters of the lesions were 0.8–14.2 cm, with an average of 5.0 ± 2.6 cm.

The final diagnosis indicated that there were eight pseudocysts (six confirmed by comprehensive diagnosis and two by surgery), nine SCNs (seven confirmed by surgery and two by comprehensive diagnosis), 13 mucinous cystic neoplasms (MCNs; 12 confirmed by surgery and one by comprehensive diagnosis), three IPMNs (two confirmed by surgery and one by comprehensive diagnosis), nine solid pseudopapillary tumors (SPTs; confirmed by surgery), six neuroendocrine tumors (five confirmed by surgery and one by biopsy), six pancreatic carcinomas (five confirmed by surgery and one by biopsy), three cysts (confirmed by comprehensive diagnosis), one inflammatory myofibroblastic tumor combined with cystic degeneration (confirmed by surgery), one solitary fibrous tumor (confirmed by surgery), and one pancreatic nerve sheath tumor (confirmed by surgery). There were also four lesions considered to be benign by comprehensive diagnosis.

The classification diagnosis results are shown in Table 1.

Each type corresponded to several certain kinds of cystic pancreatic diseases. Type I pseudocysts and cysts accounted for 61.1% (11/18), type II SCNs accounted for 71.4% (5/7), type III MCNs accounted for 80% (8/10), and type IV included a variety of pathologic types, typically SPTs, neuroendocrine tumors, and pancreatic carcinomas.

The sizes and positions of the lesions of each type are shown in Table 2.

The average size of type II had significant difference compared with other types (II versus I, *P* = 0.039; II versus III, *P* = 0.002; II versus IV, *P* = 0.001). The average size between types I and IV also had significant difference (I versus IV, *P* = 0.028).

### 3.2. Comparison of Diagnostic Results by US and CEUS with CT Diagnosis

The comparison between the results of US and enhanced CT is shown in Table 3. There were significant differences in the distribution of classification diagnosis results between enhanced CT and US (*P* = 0.001).

The comparison between the results of CEUS and enhanced CT is shown in Table 4. There were no significant differences in the distribution of classification diagnosis results between enhanced CT and CEUS (*P* > 0.05).

The coincidence rates of diagnosis of US and CEUS were 60.94% (39/64) and 84.38% (54/64), respectively.

A diagnostic agreement between US and enhanced CT with the kappa value was 0.36 (95% CI: 0.20–0.52), and the kappa value between CEUS and enhanced CT was 0.77 (95% CI: 0.63–0.89). The classification diagnosis results of CEUS showed substantial agreement with enhanced CT.

## 4. Discussion

Cystic pancreatic lesions are not uncommon in clinical practice. Cystic pancreatic lesions are divided into cystic tumors and cystic nonneoplastic lesions. The common types of the former include SCNs, MCNs, and IPMNs; the less common types are neuroendocrine tumors, cystic ductal adenocarcinomas, and SPTs. Cystic nonneoplastic lesions are congenital (cystic associated with von Hipple-Lindau disease, et al.) or acquired (pseudocysts and parasitic cysts) [6]. The development of imaging techniques has greatly benefitted the detection of cystic pancreatic lesions. Various types of cystic pancreatic lesions differ in terms of clinical treatment and prognosis. An accurate preoperative diagnosis will be conducive to the proper selection of treatment. US can detect cystic lesions with sensitivity, but the qualitative diagnostic accuracy is low. CEUS can more clearly show blood perfusion within the lesions, thus providing more information for the diagnosis. Previous study showed that quantitative analysis of the enhancement of the cystic wall may discriminate the different types of the cystic pancreatic lesions [7]. CEUS can differentiate between pseudocysts and cystic tumors with accuracy [8]. Rickes and Wermke [9] found 95% sensitivity and 92% specificity in the diagnosis of cystadenoma and 100% sensitivity and specificity in the diagnosis of pseudocyst in 31 patients with cystic pancreatic masses.

However, some cystic lesions present similar morphologic features, which make the preoperative imaging diagnosis difficult. In this study, a simple classification diagnostic criterion was applied. Various types corresponding to different diseases have certain regularity. Type I lesions are unilocular cysts. Pseudocysts are the most common type I. A study showed that morbidity or mortality due to the small unilocular cysts which were ≤2 cm in size is extremely unlikely, and observation appears to be a safe management option [6]. Type II is more common in SCN. SCNs are benign tumors and in asymptomatic patients often do not require surgical resection [10]. Type III includes MCN and IPMN. At present, all MCNs are considered at least potentially malignant and all surgically fit patients should undergo surgical resection [11, 12]. IPMNs can be classified as main duct, branch duct, or mixed IPMNs. All main-duct type IPMN should be resected because of the high malignancy rate whereas branch-duct type IPMN demonstrating favourable features (<3 cm size and absence of mural nodules) may be managed conservatively [12]. Type IV includes cystic tumors as well as solid pancreatic tumors associated with a cystic component or cystic degeneration (neuroendocrine tumors, SPTs, etc.). Tumors of this type are either malignant or having malignant potential; surgical resection is the accepted method of management [13].

**Figure 2:**
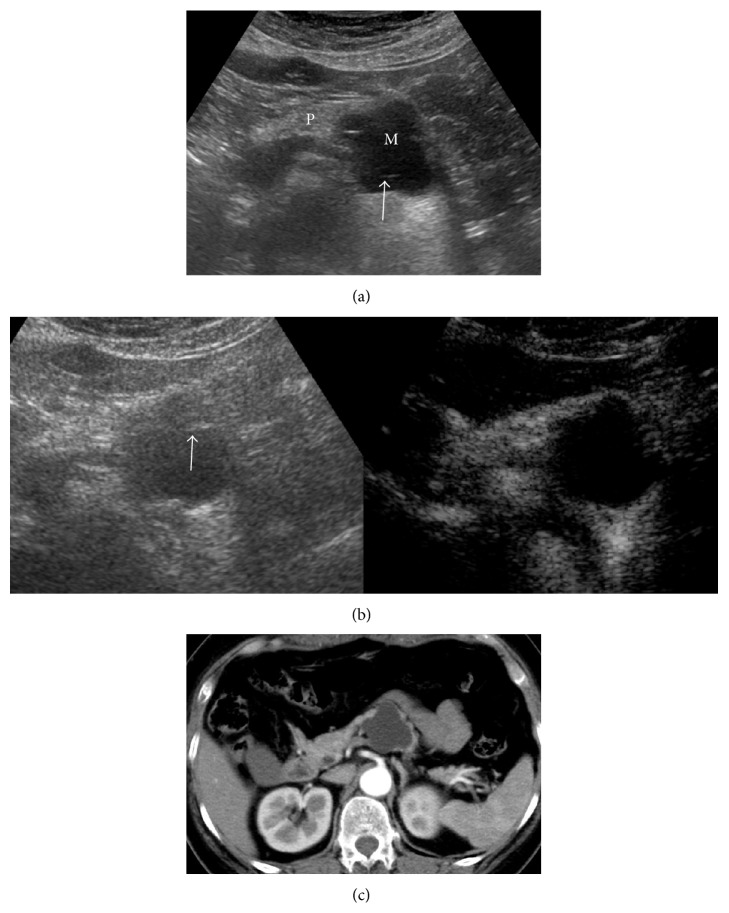
Pancreatic lesion was found by physical examination and was diagnosed as MCN by surgical pathology. (a) Cystic lesions in the tail of the pancreas were indicated by US (M: mass; P: pancreas), with multiple septa (arrow). The case was diagnosed as type III. (b) Enhancement was not shown in cystic lesions in CEUS (the right picture). The case was diagnosed as type I. (c) Enhanced CT indicated no enhancement in the cystic lesion. The case was diagnosed as type I.

**Figure 3:**
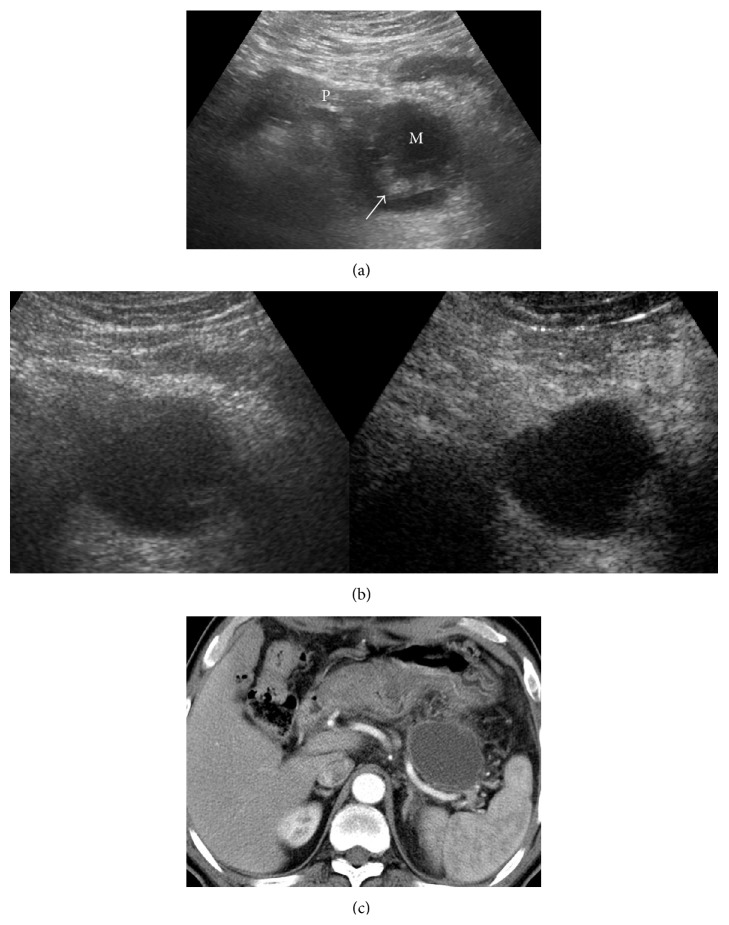
Pancreatic lesion was found by follow-up after acute pancreatitis. The case was diagnosed as a pseudocyst by surgical pathology. (a) US indicated cystic lesion in the tail of the pancreas (M: mass; P: pancreas), which contained low-echo solid components (arrow). The case was diagnosed as type IV by US. (b) There was no enhancement inside the cystic lesion in CEUS (the right picture). The case was diagnosed as type I by CEUS. (c) Enhanced CT indicated no enhancement in the cystic lesion. The case was diagnosed as type I.

Applying the classification diagnostic criterion, the diagnostic value of CEUS in cystic pancreatic lesions was discussed compared with the contrast-enhanced CT. As reported herein, the coincidence rate of diagnosis by CEUS was higher than US for cystic pancreatic lesions (84.38% versus 60.94%). The CEUS results agreed well with the enhanced CT results (kappa = 0.77). The contrast agent, SonoVue, is a blood pool contrast agent, which is distributed entirely within the blood vessels. This contrast agent can dynamically reveal the blood supply in the lesions and the microvessel structure in real time. Both the temporal and spatial resolutions are high. The study showed that CEUS could clearly show the septa and mural nodules in the lesions. Moreover, the presence of solid components, necrosis, and mucus in the lesions was detected by whether or not the echoes were enhanced (Figures 2 and 3).

There were a few cases which had inconsistent diagnosis between CEUS and CT. This possibly occurred by the insufficient assessment of the septa and mural nodules at CEUS due to the obesity of patient and deep location of lesion. For some larger lesions, incomplete scanning at CEUS may also affect the diagnosis.

**Figure 4:**
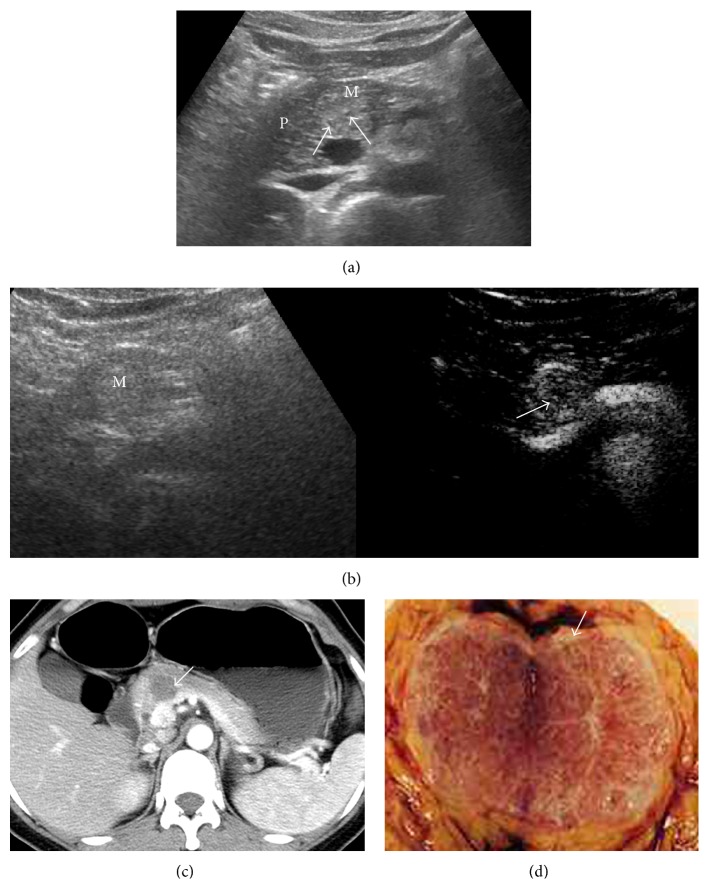
Pancreatic lesion was detected by physical examination. (a) US indicated a hyperechoic lesion in the pancreatic neck (M: mass; P: pancreas). The multiple microcysts were observed (arrows). The case was diagnosed as type IV by US. (b) The lesions showed heterogeneously enhancement in CEUS (the right picture). There were small cystic nonenhancement areas in the lesion (arrow). The case was diagnosed as type IV by CEUS. (c) Enhanced CT indicated no enhancement in the majority of the lesion. The fine septa enhancement could be seen (arrow) and type II was suspected by CT. (d) The surgical specimen was observed with a honeycomb shape with multiple compartments in the lesions. The case was pathologically diagnosed as microcystic SCNs.

Type II cases in our group were mostly SCNs, with the imaging features demonstrating multilocular and microcysts (cavity diameter < 2 cm). The radial-shaped calcification in the middle can be used as the typical feature of a SCN [14]. Under the microscope, the cystic wall of the SCN is composed of simple cuboidal epithelial cells. The fibrous septum between the tumor cells is rich in microvessels [15]. The lesion showed higher enhancement in the early stage of CEUS, which was similar to enhanced CT. The SCN, with a small cystic cavity, intensive septa, and microcysts, is likely to be confused with solid lesions by US or CEUS. In our group, two patients with SCNs with microcysts showed round, clearly defined, and hyperechoic lesions on US. In CEUS, the intensive septa in the lesions were clearly enhanced with nonenhancement small cavities and the performance was similar to solid components. These cases were misdiagnosed as type IV by US and CEUS. The surgical specimen showed the honeycomb pattern composed of multiple microcysts with a diameter of several millimeters (Figure 4).

There were many pathologic subtypes of type IV demonstrated in the current study, such as SPTs, neuroendocrine tumors, and pancreatic carcinomas. Of nine patients with SPTs, seven (77.8%) had a circular enhancement capsule in the early stage of CEUS, which was consistent with a previous report [16]. Pancreatic carcinoma is hypovascular, while the neuroendocrine tumor is hypervascular. The solid components are hypo- and hyperenhanced in the early stage of CEUS, respectively. This feature can be used for differentiation between the two types of tumors.

The mean sizes of type I and II lesions were 3.9 ± 2.1 cm and 2.3 ± 0.6 cm, respectively. Type III and type IV lesions were larger, with an average size of 6.3 ± 2.4 cm and 6.0 ± 2.6 cm, respectively. Previous study [17] showed that the PPV of cystic lesions <3 cm to the diagnosis of benign lesions was 87%. Of 35 cases of unilocular cysts without septa and mural nodules, 34 cases were benign and only one was a borderline tumor. Among patients with compartments, 80% were benign. The study proposed that most of the cystic pancreatic lesions <3 cm in diameter were benign, and the patients can be followed up selectively. Our study also indicated that type I and II lesions were mostly pseudocysts and SCNs, both of which are benign lesions. Therefore, for different types of cases, the size of the lesion is an important consideration for the selection of clinical treatment.

There were several limitations to the current study. First, for patients with small cystic pancreatic lesions (<1 cm) and located in a deeper position, it is sometimes difficult to judge whether or not the inside of the lesions is enhanced. Hypoenhancement may occur. However, the constant degree of enhancement from the early to the late stage of the scan may indicate no enhancement. Second, not all the patients had pathological diagnosis. The cases with higher benign probability were confirmed by typical imaging findings and comprehensive diagnosis in follow-up. Further study may be required which takes pathology as the criterion.

Cystic pancreatic lesions are varied in pathologic types. The diagnostic accuracy of CEUS based on the morphologic features was greater than US. Some tumors exhibited typical features in CEUS, which benefits the preoperative diagnosis and selection of treatment. As an economic, radiation-free, and effective imaging technique, CEUS can be considered as a supplement examination for the qualification of cystic pancreatic lesions and be used as a follow-up technique.

## Figures and Tables

**Table 1:** Classification of cystic pancreatic lesions by CT in 64 cases.

Final diagnosis	Number	I	II	III	IV
Pseudocyst	8	8	0	0	0
SCN	9	2	5	1	1
MCN	13	1	0	8	4
IPMN	3	0	0	1	2
SPT	9	0	0	0	9
Neuroendocrine tumor	6	1	0	0	5
Pancreatic carcinoma	6	0	0	0	6
Cyst	3	3	0	0	0
Other	7	3	2	0	2

Total	64	18	7	10	29

**Table 2:** The sizes, locations, and calcifications of the lesions of each type.

Type	Number	Diameter (cm)	Location				Calcification
		(mean ± SD)	Head	Body	Tail	Multiple	
I	18	0.8–8.0 (3.9 ± 2.1)	4	4	9	1	1
II	7	1.4–3.2 (2.3 ± 0.6)	2	3	1	1	1
III	10	3.0–11.0 (6.3 ± 2.4)	3	3	4	0	2
IV	29	2.7–14.2 (6.0 ± 2.6)	11	8	9	1	6

**Table 3:** Comparison between the results of US and enhanced CT.

		US				Total
		I	II	III	IV	
Enhanced CT	I	6	0	2	10	18
	II	0	3	0	4	7
	III	0	1	2	7	10
	IV	0	1	0	28	29

Total		6	5	4	49	64

**Table 4:** Comparison between the results of CEUS and enhanced CT.

		CEUS				Total
		I	II	III	IV	
Enhanced CT	I	14	0	3	1	18
	II	0	5	0	2	7
	III	1	0	8	1	10
	IV	0	1	1	27	29

Total		15	6	12	31	64
